# Does influenza vaccination protect people with breakthrough infections from acute cardiovascular events?

**DOI:** 10.2807/1560-7917.ES.2026.31.13.2600281

**Published:** 2026-04-02

**Authors:** Jeffrey C Kwong

**Affiliations:** 1 ICES, Toronto, Ontario, Canada; 2 Public Health Ontario, Toronto, Ontario, Canada; 3 Department of Family & Community Medicine, University of Toronto, Toronto, Ontario, Canada; 4 Dalla Lana School of Public Health, University of Toronto, Toronto, Ontario, Canada; 5 University Health Network, Toronto, Ontario, Canada

**Keywords:** laboratory-confirmed influenza, myocardial infarction, stroke, influenza vaccination, self-controlled case series

Medical advances and health promotion efforts have substantially reduced the incidence of cardiovascular events such as acute myocardial infarction (AMI) and stroke, in recent decades [1]. Yet, cardiovascular disease remains one of the top causes of disease burden worldwide [1], with influenza virus infection being recognised as an important trigger of cardiovascular events [2]. This means that non-trivial proportions of AMI and stroke could be prevented by vaccination. Indeed, randomised controlled trials have demonstrated that influenza vaccination reduces short-term cardiovascular mortality and events [3], making these vaccines a powerful tool for preventing important sequelae of influenza virus infections. However, whether the reduction in cardiovascular mortality and events is achieved by reducing the *incidence* or *severity* of influenza virus infections is uncertain.

This issue of *Eurosurveillance* features a study in which Croci et al. sought to confirm the association between influenza virus infection and AMI and stroke, and to determine if influenza vaccination could reduce the risk of these short-term cardiovascular events despite breakthrough infections among vaccinated individuals. In other words, the authors investigated if in cases where influenza vaccination did not prevent infection, whether vaccination could still prevent that individual from experiencing a cardiovascular event by mitigating the severity of the infection [4].

A previous study by our team that was primarily focused on establishing the association between laboratory-confirmed influenza virus infection and AMI investigated 364 episodes of AMI among individuals who also had laboratory-confirmed influenza [5]. In a subgroup analysis, the incidence rate ratio (IRR) for the association between influenza virus infection and AMI was 5.66 (95% confidence interval (CI): 2.49–12.87) for vaccinated individuals and 6.24 (95% CI: 3.64–10.70) for unvaccinated individuals. The p value for the interaction term for this subgroup analysis was 0.85, indicating no difference in the exposure-outcome association between the two groups. In the study by Croci et al., which included 1,231 cardiovascular episodes (792 strokes, 429 AMI), the IRR was lower among vaccinated individuals (2.4; 95% CI: 1.5–3.8) compared with unvaccinated individuals (4.7; 95% CI: 3.3–6.6) [4]. The ratio of the two IRRs was 0.51 (95% CI: 0.29–0.91; interaction p = 0.020), suggesting that vaccination may reduce the severity of infection – measured in terms of reduced risk of short-term cardiovascular events – among those with breakthrough influenza virus infection.

While this finding is promising indeed, deeper examination is warranted. Could it be simply due to differences between the vaccinated and unvaccinated populations i.e. confounding bias? Although the self-controlled case series design applied in the study inherently controls for all time-invariant confounders, such as sex, ethnicity, and socioeconomic status, by using cases as their own controls [6], the observed difference in IRRs between vaccinated and unvaccinated individuals does not account for potential differences in characteristics between the two groups. Vaccinated and unvaccinated individuals may differ in important ways, such as the presence of cardiovascular disease risk factors and engagement in health-promoting behaviours. However, Figure 3A in the article is reassuring, as it shows that the point estimates of the IRRs differed only during days 1–3 and 4–7 after influenza virus infection, and not during the subsequent risk periods spanning 8–90 days, suggesting that the observed difference in IRRs in the two groups is specific to influenza virus infection. Furthermore, there was no observed difference in the IRRs between the vaccinated and unvaccinated groups for the negative control exposure of *Campylobacter* spp. infection. Regardless, confirmation of this promising finding will be important to broaden the evidence base, and future studies using similarly large (or larger) sample sizes should attempt to replicate this analysis, while ensuring that any differences between groups are accounted for. Preferably, this should also be done for AMI and stroke separately as done by Croci et al.

Irrespective of the mechanism of how influenza vaccination prevents cardiovascular events, the unfortunate reality is that influenza vaccine coverage among patients with cardiovascular disease generally remains well below the 75% target for people with chronic conditions that was set by the World Health Organization, with available recent estimates from countries such as Denmark, Korea and the United States in the range of 40-44% [7-9]. Efforts are needed to increase influenza vaccine coverage in this high-risk population. In particular, addressing misperceptions of low risk of influenza and concerns about the safety and effectiveness of influenza vaccination will require education of both patients and providers, and strong recommendations for vaccination from healthcare providers [10]. A number of system-level measures have been shown to increase influenza vaccine coverage in patients with cardiovascular disease; these include making influenza vaccines available at no cost, offering financial incentives, implementing patient reminder-recall and provider feedback-audit systems, sending electronic nudge letters, and taking every available opportunity to offer influenza vaccines to patients during interactions at healthcare settings (e.g. physician offices, pharmacies, emergency departments, hospitals) and even non-healthcare settings (e.g. workplaces) [7,10]. For those aged 65 years or older, high-dose (or other enhanced) influenza vaccines could be offered based on their increased effectiveness in preventing cardiorespiratory hospitalisations [11,12]. Ideally, even more effective and cross-protective influenza vaccines should be developed. High-risk individuals – especially those not accepting vaccination – should be advised to engage in healthy behaviours such as washing hands, wearing masks, getting adequate sleep, and participating in regular physical activity as much as possible, to protect themselves from respiratory infections [13-16]. Moving forward, a combination of all these measures will contribute to reducing cardiovascular events due to respiratory pathogens such as influenza viruses.

## Note

Dr Jeffrey C Kwong is a scientist at both ICES and Public Health Ontario, and a professor of family medicine and public health at the University of Toronto. He leverages population-based health databases to study the epidemiology of infectious diseases and to generate real-world evidence for optimising vaccination programmes.
